# Outcome measures in Angelman syndrome

**DOI:** 10.1186/s11689-024-09516-1

**Published:** 2024-03-01

**Authors:** Doesjka A. Hagenaar, Karen G. C. B. Bindels-de Heus, Maud M. van Gils, Louise van den Berg, Leontine W. ten Hoopen, Philine Affourtit, Johan J. M. Pel, Koen F. M. Joosten, Manon H. J. Hillegers, Henriëtte A. Moll, Marie-Claire Y. de Wit, Gwen C. Dieleman, Sabine E. Mous

**Affiliations:** 1 ENCORE Expertise Centre for Neurodevelopmental Disorders, Erasmus MC, Rotterdam, The Netherlands; 2 Department of Child- and Adolescent Psychiatry/Psychology, Erasmus MC, Rotterdam, The Netherlands; 3 Department of Paediatrics, Erasmus MC, Rotterdam, The Netherlands; 4 Vestibular and Oculomotor Research Group, Department of Neuroscience, Erasmus Medical Center, Rotterdam, The Netherlands; 5 Department of Dietetics, Erasmus MC, Rotterdam, The Netherlands; 6 Division of Pediatric ICU, Department of Neonatal and Pediatric ICU, Erasmus MC, Rotterdam, The Netherlands; 7 Department of Neurology and Paediatric Neurology, Erasmus MC, Rotterdam, The Netherlands

**Keywords:** Angelman syndrome, Outcome measures, Eye-tracking, Functional near-Infrared Spectroscopy, Indirect calorimetry, Bio-impedance analysis, BOD POD

## Abstract

**Background:**

Angelman syndrome (AS) is a rare neurodevelopmental disorder characterized by severe intellectual disability, little to no expressive speech, visual and motor problems, emotional/behavioral challenges, and a tendency towards hyperphagia and weight gain. The characteristics of AS make it difficult to measure these children’s functioning with standard clinical tests. Feasible outcome measures are needed to measure current functioning and change over time, in clinical practice and clinical trials.

**Aim:**

Our first aim is to assess the feasibility of several functional tests. We target domains of neurocognitive functioning and physical growth using the following measurement methods: eye-tracking, functional Near-Infrared Spectroscopy (fNIRS), indirect calorimetry, bio-impedance analysis (BIA), and BOD POD (air-displacement plethysmography). Our second aim is to explore the results of the above measures, in order to better understand the AS phenotype.

**Methods:**

The study sample consisted of 28 children with AS aged 2–18 years. We defined an outcome measure as feasible when (1) at least 70% of participants successfully finished the measurement and (2) at least 60% of those participants had acceptable data quality. Adaptations to the test procedure and reasons for early termination were noted. Parents rated acceptability and importance and were invited to make recommendations to increase feasibility. The results of the measures were explored.

**Results:**

Outcome measures obtained with eye-tracking and BOD POD met the definition of feasibility, while fNIRS, indirect calorimetry, and BIA did not. The most important reasons for early termination of measurements were showing signs of protest, inability to sit still and poor/no calibration (eye-tracking specific). Post-calibration was often applied to obtain valid eye-tracking results. Parents rated the BOD POD als most acceptable and fNIRS as least acceptable for their child. All outcome measures were rated to be important. Exploratory results indicated longer reaction times to high salient visual stimuli (eye-tracking) as well as high body fat percentage (BOD POD).

**Conclusions:**

Eye-tracking and BOD POD are feasible measurement methods for children with AS. Eye-tracking was successfully used to assess visual orienting functions in the current study and (with some practical adaptations) can potentially be used to assess other outcomes as well. BOD POD was successfully used to examine body composition.

**Trial registration:**

Registered d.d. 23-04-2020 under number ‘NL8550’ in the Dutch Trial Register: https://onderzoekmetmensen.nl/en/trial/23075

## Background

Angelman syndrome (AS) is a rare neurogenetic disorder characterized by severe intellectual disability, little or no expressive speech, and motor difficulties. Children with AS frequently have epilepsy, sleeping problems, and behavioral issues such as hyperactivity, attention problems, and anxiety [1]. The symptoms of AS are caused by a loss-of-function of the UBE3A gene on the maternal chromosome 15q11-q13. This loss-of-function is the result of a chromosomal microdeletion (60–70%), a pathogenic variant of the UBE3A gene (15%), a uniparental paternal disomy (UPD; 5–10%), or an imprinting center defect (ICD; 5–10%) [2].

Some of the characteristics of AS make it difficult to measure functioning in these children. Commonly used tests for children with AS often rely on a certain level of cognitive, attentional, communicative, and motor skills. Test instructions are not always understood by a child with AS. If a child with AS knows a certain answer, it may be impossible for them to communicate this due to problems with expressive language or motor functioning. Behavioral issues such as hyperactivity, attention problems, anxiety, and sensitivity to sensory stimuli can interfere with taking a measurement or impact validity of the results. As a consequence of the above, suitable outcome measures are scarce for children with AS. Since suitable outcome measures allow us to measure the current functioning of a child with AS, and possible changes over time, they are necessary and of great importance. In clinical practice, this will result in better identification of the child’s strengths and difficulties and earlier identification of health problems. It will also allow evaluation of the effect of interventions. Ultimately, this will lead to improved guidance and treatment of the child. In scientific research, the availability of suitable outcome measures will facilitate in-depth phenotyping. In addition, outcome measures are important for clinical trials to measure the potential effects and side effects of new treatments. Phase 1/2 clinical trials are currently underway to measure the safety of an antisense-oligonucleotide treatment in people with AS (e.g., [3]). This is the first treatment targeting the cause of AS, and pre-clinical studies have shown promising results in a mouse model [4]. Future clinical trials are expected to investigate the effectiveness of these and other treatments. Therefore, there is an urgent need for suitable outcome measures in AS, as supported by other studies [5, 6]. Previous initiatives in the search for suitable outcome measures focused on outcome domains such as gait [7, 8], sleep/EEG [6, 8–10], and communication [6, 11, 12].

The current study examined five novel measures of neurocognitive functioning and physical growth in children with AS that may lead to creative and out-of-the-box solutions, namely:

- Eye-tracking
- Functional Near-Infrared Spectroscopy (fNIRS)
- Indirect calorimetry
- Bio-impedance analysis (BIA)
- Whole body air-displacement plethysmography (BOD POD)

### Eye-tracking

Eye-tracking is a noninvasive instrument to measure gaze location on a screen. It is a promising measurement tool for children with AS, as it does not rely on understanding test instructions or verbal communication of the answer, nor does it require the child to push a button or make a motor response (other than eye-movements). The technique can be used to measure a variety of outcomes depending on the task paradigm provided. Three recent eye-tracking studies have been conducted in individuals with AS, measuring social attention [13, 14], and language comprehension [15]. Success rates were highly variable, ranging from 47 to 83 percent, and data quality was not reported. In the current study, eye-tracking was used to measure (1) visual orienting functions and (2) social preference. Orienting responses to basic visual stimuli (1), such as contrast, form, and motion, were measured to quantify the first steps in visual processing [16]. Adequate orienting responses are a prerequisite for guiding visual attention to new features in one’s environment. It was shown in a previous study that children with AS have impairments in basic visual functions [17]. When integrated in a larger functional brain network, such as the what and where pathways [18], visual processing becomes the starting point of perception, visually augmented communication (e.g., pictograms), and social interactions. Second, we tested social preference (2), namely, preference for faces in comparison to non-social stimuli. The effect that faces capture and maintain children’s attention more than non-social stimuli has been shown to exist from as early as 6 months of age in the general population [19].

### Functional Near-Infrared Spectroscopy (fNIRS)

fNIRS is a safe and non-invasive neuroimaging method to measure cortical activity using only a wireless head cap. For children with AS, laying still in a noisy and closed MRI scanner is nearly impossible, and the only way to conduct an MRI scan is under general anesthesia (e.g., [20]). fNIRS offers a non-invasive alternative to fMRI and is therefore a promising measurement method for children with AS. Unlike fMRI, fNIRS measurements are silent (do not make noise) and can take place at almost every location. There is no need for head fixation or lying still on a bed. fNIRS paradigms can be “passive” in the sense that they do not rely on language and motor functioning, nor do they require a specific level of intellectual functioning. fNIRS has successfully been applied in children with attention-deficit/hyperactivity disorder (ADHD) [21], autism spectrum disorder (ASD) [22], prenatal alcohol abuse [23], and Down syndrome [24], but has not yet been evaluated in AS. The outcome measured with fNIRS is cortical activation in response to a social paradigm. This paradigm was previously proven to elicit a robust cortical response in the superior temporal sulcus in typically developing children and was able to differentiate between infants at risk for ASD and low-risk controls [25–28].

### Indirect calorimetry, bio-impedance analysis (BIA), and BOD POD

In this study, indirect calorimetry was used to measure resting metabolic rate (RMR; the energy used to sustain vital functions at rest), while BIA and BOD POD were used to measure body composition (fat and fat-free mass). Overweight and obesity occur in approximately 40% of children with AS [29]. Outcome measures on body composition and RMR in children with AS are needed to identify children with high bodyfat (at risk for secondary health issues) and to give individualized nutrition advice based on measured RMR. In addition, outcome measures on metabolism and fat percentage are important for research on the pathogenesis of overweight and obesity in AS. Finally, they could serve as outcome measures for treatment studies, as restoring UBE3A function rescues the overweight seen in AS mice models [30, 31].

### Aim

The first aim of this study is to test the feasibility of eye-tracking, fNIRS, indirect calorimetry, BIA, and BOD POD in children with AS. Each measure was evaluated in terms of success rate (successfully finished yes/no) and data quality. In addition, we described reasons for early termination, adaptations to measurement procedures, recommendations given by parents, and acceptability as rated by parents. The second aim of this study was to explore the results of the above measures, in order to better understand the AS phenotype.

## Methods

### Participants

**Fig. 1:**
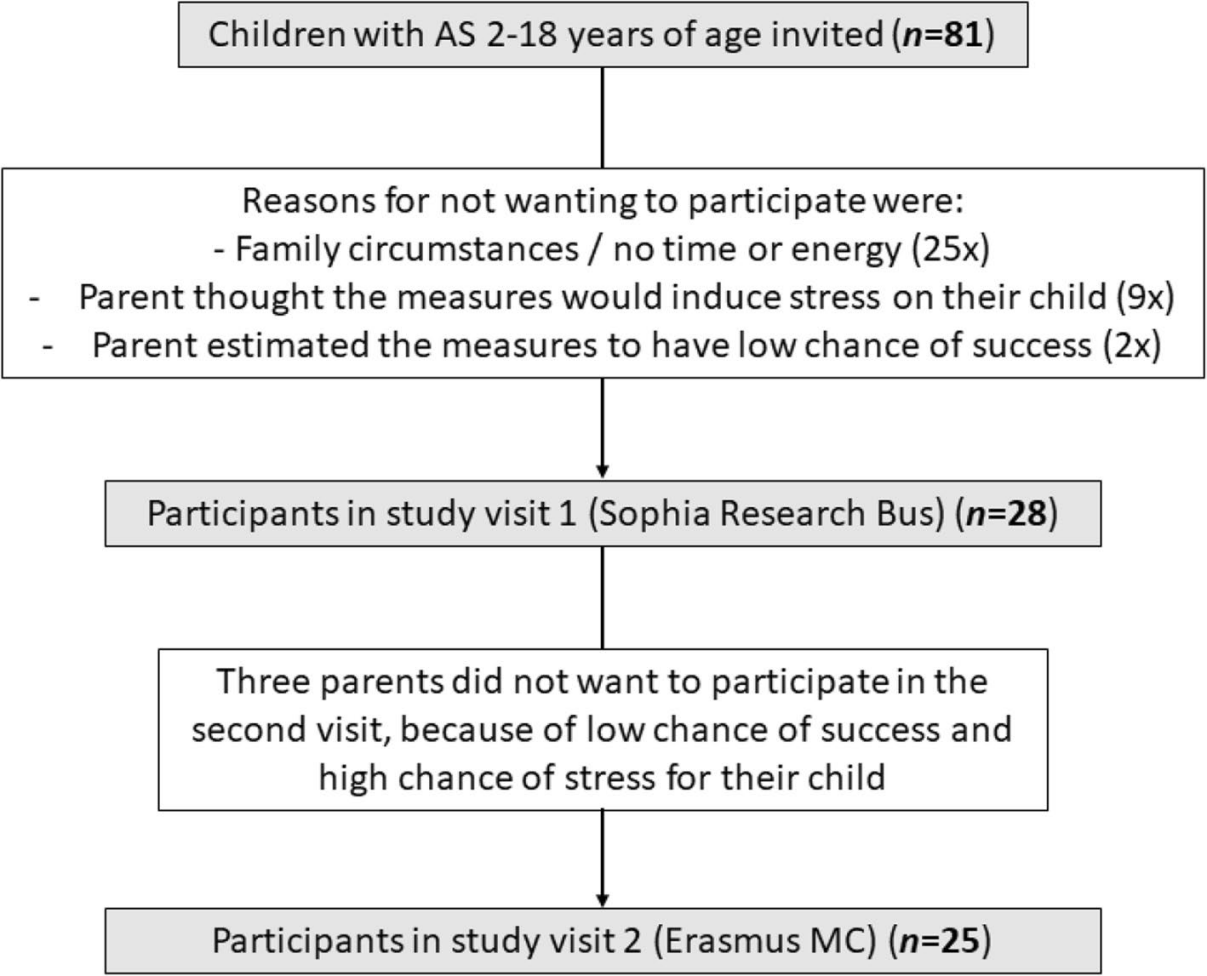
Flow chart of (potential) participants

All patients under care of the Expertise Centre for AS at the Erasmus Medical Centre Sophia Children’s Hospital were invited to participate in this study. Inclusion criteria for the child were having an age between 2 and 18 years and having a genetically confirmed diagnosis of AS (details on the process of molecular diagnostics can be found in reference [29]). An inclusion criterion for the parent/caregiver was having an adequate understanding of the Dutch or English language. Exclusion criteria for the child were having a current non-convulsive status epilepticus or inter-current somatic illness influencing daily functioning, the presence of a mosaic form of AS, or current participation in a disease-modifying treatment study. In total, 81 children with AS were invited to participate in this study. One-third of them participated. Figure 1 shows the flow chart of (potential) participants and the reasons for not participating. The current study sample consisted of 28 participants. Demographical characteristics are shown in Table 1. Participants did not differ significantly from non-participants on age (*T* (79) = 0.33, *p* = 0.741), sex (*Χ*^2^ (1) = 0.16, *p* = 0.686), or genotype (*Χ*^2^ (3) = 2.89, *p* = 0.410).

**Table 1:** Demographical and phenotypical characteristics of the study sample (*n* = 28)

	**Frequency**
**Sex**	
Male	14
Female	14
**Genotype**	
Deletion	15
Non-deletion	13
*Paternal uniparental disomy (UPD)*	*7*
*Imprinting center defect (ICD)*	*1*
*UBE3A mutation*	*5*
**Epilepsy**	
Yes	22
*Yes, active*	*16*
*Yes, controlled with anti-seizure medication*	*6*
No	6
*No, never*	*4*
*In remission (currently no anti-seizure medication)*	*2*
**Socio-economic status**^a^	
Low-level education^b^	1
Middle-level education^c^	16
High-level education^d^	11
**Comorbid psychiatric diagnoses**	
No psychiatric diagnosis	21
Attention deficit hyperactivity disorder (ADHD)	6
Autism spectrum disorder (ASD)	1
**Medication use**	
Antiseizure medication	
*Valproic acid*	13
*Clobazam*	*10*
*Levetiracetam*	*6*
*Topiramate*	*3*
*Diazepam, lamotrigin, or ethosuximide*	*2*
*Ethosuximide, gabapentine, clonazepam, lacosamide, brivaracetam, or rufinamide*	*1*
Psychotropic medication	
*Methylphenidate*	*4*
*Risperidone*	*2*
*Aripiprazol*	*1*
No medication	5
**Vision (as reported by parents)**	
Normal vision	9
Farsighted^e^	9
Strabismus	8
No stereovision	5
Nearsighted^e^	4
Nystagmus	2
**Walking abilities**	
Walking independently	18
Walking with support	7
Wheelchair dependent	3
	**Mean (SD)**
**Age**	11.04 (4.51)
**CGI-S score**^**f**^	
Total	4.68 (0.78)
Behavior	4.00 (0.98)
Motor (fine/gross)	4.68 (0.61)/4.29 (1.33)
Communication (expressive/receptive)	4.93 (1.05)/3.50 (1.17)
Sleep	3.46 (1.64)
**PEDI-CAT scaled scores (*N* = 20)**^**g**^	
Daily activities	47.58 (4.17)
Mobility	58.26 (5.28)
Social/cognitive	53.89 (4.62)

*Abbreviations*: *CGI-S* Clinical Global Impression of Severity Scale; *PEDI-CAT* Pediatric Evaluation of Disability Inventory Computer Adaptive Test

^a^Defined as highest education (average) of both parents

^b^Low-level education consisted of: no education or primary education only

^c^Middle-level education consisted of: secondary education only or middle-level vocational education

^d^High-level education consisted of: high level vocational education, university education, or PhD education

^e^Only one participant tolerated wearing glasses, while 12 others were supposed to wear glassed but did not tolerate them

^f^The CGI was adapted to the AS population by the authors. A score of 1 represents normal development, 2 is slightly impaired, 3 is mildly impaired, 4 represents moderately impaired, 5 is markedly impaired, 6 is severely impaired, and 7 represents very severe impairments (among the most extremely impaired)

^g^Scaled scores on the PEDI-CAT lay on a continuum of 20 (low function) to 80 (high function) and are not age-related. *T*-scores were not reported, as more than half of the population had a *T*-score lower than 10, under which no differentiation was possible

### Procedure

The Rotterdam Outcome Study for children with Angelman syndrome (ROSA) was an experimental cross-sectional study conducted by the Expertise Centre for AS (part of ENCORE Expertise Centre for Neurodevelopmental Disorders) at the Erasmus MC Sophia Children’s Hospital in Rotterdam, the Netherlands. This study was approved by the medical ethical board of the Erasmus MC (MEC-2020-0489). All participants (their legal representatives) signed informed consent. Data was collected between May 2021 and August 2022.

### Preparation of study visits

Before the study visits, parents were sent a leaflet with pictograms to inform and prepare their child for the study visits. Further, a “practice fNIRS cap” (swimming cap) was sent home. During a phone call, the study procedure were explained, suggestions on how to prepare their child were given, and any questions were answered.

#### Study visit 1: eye-tracking and fNIRS

The first study visit took place in the Sophia Research Bus [32], a campervan especially equipped for scientific research. The Sophia Research Bus contains a desk with the fNIRS and eye-tracking set up and is made accessible for children in a wheelchair using a lift. The researcher drove the Sophia Research Bus to a location of the parent’s choosing, allowing us to visit all participants close to their home or school, minimizing the burden of participation while still having the benefit of performing the assessment in a standardized environment. In addition, not needing to travel minimized potential fatigue and sensory overload at the moment of testing of the participants, thereby facilitating them to perform the tests to the best of their abilities. One researcher performed all measurements, in the presence of a parent/caregiver. The total duration of the measurements in the Sophia Research Bus was 30 to 45 min.

#### Study visit 2: indirect calorimetry, BIA, and BOD POD

The indirect calorimetry, BOD POD, and BIA measurements were conducted during a second study visit to the Erasmus MC Sophia Children’s Hospital. Participants did not eat or drink for at least 2 h before the measurements. They were also instructed to refrain from physical effort during this period. A dietician performed the measurements, while the researcher and parent/caregiver were present for additional support. The total duration of this second study visit was 2 to 3 h, but this also included breaks and several measurements that were not part of the current study.

## Measures

### Eye-tracking

Gaze location was measured using a Tobii TX300 eye-tracker with a 23″ screen (Tobii Corporation, Danderyd, Sweden) and a five-point calibration procedure. This eye-tracker uses video sensors to assess the location of the pupil center and the centers of reflections of one or several near-infrared illuminators. Participants were required to sit on a chair facing the screen, paying attention to the screen and sitting relatively still. The Visual Orienting Functions (VOF) task was developed by Kooiker et al. [16] (see original article for full description) and lasted approximately 7 min. Tobii Studio was used to display a set of images and movies to measure basic visual processing functions: form coherence processing, local motion processing, global motion processing, contrast detection, and color detection. In addition, a high salient cartoon stimulus presented all different types of visual information at once, to measure the efficiency of simultaneous visual information processing. The social attention task was developed by Gliga et al. [19] (see original article for full description) and had a duration of 2 min. E-prime 2 and E-prime Extensions for Tobii 2 were used to assess attention capture and sustained attention to faces in comparison to non-social stimuli. Additional information on the eye-tracking measures is given in Appendix 1.

### Functional Near-Infrared Spectroscopy (fNIRS)

Changes in hemodynamic response signal in the superior temporal sulcus region were measured using the Brite 23 with Oxysoft software (Artinis Medical Systems, 2017). The working mechanism behind fNIRS is its use of near-infrared light through optodes on the scalp, which is differentially absorbed by oxy- and deoxygenated hemoglobin. The optodes were attached to a tight headcap. Participants wore the cap for 10 to 15 min, during which they sat on a chair facing a screen. Light counts were optimized by removing hair underneath the optodes using a pointed comb. A video-based method was used to estimate the position of fNIRS optodes on the scalp [33]. A passive social block-design task designed by Lloyd-Fox et al. [27] was displayed by E-prime 2. The task showed dynamic visual and auditory social stimuli, contrasted to a baseline of non-social stimuli (duration approx. 6 min). A full description of the task can be found in the original paper. Appendix 1 provides additional information on the current study’s fNIRS measurement.

### Indirect calorimetry

RMR was assessed using an indirect calorimeter (Q-NRG+, Cosmed, Italy). This device analyzes the composition of the air that participants breathe in and out, i.e., how much oxygen was consumed and how much carbon dioxide was produced. Participants laid on a bench, when a plastic hood was put over their head in which they could breathe normally. The measurement took approximately 30 min. The result is most reliable if the participant is fully in rest (i.e., lays still). The measure is intended for use with subjects above 15 kg.

### Bio-impedance analysis (BIA)

Fat- and fat-free percentage was measured using an InBodyS10. Two electrodes were attached to each hand and foot using stickers or clips. Impedance was measured by a weak electrical current that cannot be felt. Participants had to sit or lay still with their arms and legs wide, not touching the torso or conductive materials. After attaching the stickers and clips, the measurement duration was approximately 1 min.

### BOD POD (air-displacement plethysmography)

A BOD POD® (COSMED USA, Inc. Concord, CA) device measured body composition by air displacement plethysmography. Participants wore tight underwear and a swimming cap. Before the measurement, participants had to stand on the scale attached to the BOD POD for 10 s (no wheelchair or balance support option). Following, participants sat in the closed BOD POD for 2 or 3 min. The measurement is most reliable when the participant sits still.

Weight and height were measured prior to the measurements. Weight was measured to the nearest 0.01 kg using calibrated scales. Standing height was obtained by a stadiometer in children who are able to stand, and in lying position with a measuring tape in children who were unable to stand.

### Data analyses

We defined a measure as feasible when (1) at least 70% of all participants that started a particular measurement, successfully finished the measurement and (2) at least 60% of the participants that finished a measurement, contributed to data of acceptable quality. As there is no golden standard for the definition of feasibility in children with intellectual disabilities, these numbers were defined in advance during an expert consensus meeting (with authors KBdH, LtH, MCdW, GD, SM, and DH), based on previous literature on eye-tracking and EEG in AS [13, 34, 35]. For the other outcome measures, no previous studies in AS were available. Furthermore, the criteria were presented to and approved by the medical ethical board (including a methodologist/statistician).

A detailed description of data quality assessment per measurement instrument can be found in Appendix 2. Additional feasibility points were as follows:

- Percentage of participants (their legal representatives) that refused to participate in this specific measurement beforehand, and the reason for refusing
- Reasons for early termination of the measurement
- Adaptations made to the measurements
- Acceptability and importance of the measure/paradigm as rated by participants’ legal representatives by three questions (rated on a ten-point scale):
  - ◦ “How much do you think your child enjoyed the measurement?”(where 0 means no enjoyment at all and 10 means much enjoyment)
  - ◦ “How stressful do you think the measurement was for your child?” (where 0 means not stressful at all and 10 means very stressful)
  - ◦ “To what extend do you think this task measures an important/relevant aspect of your child’s functioning?” (where 0 means not important/relevant at all and 10 means very important/relevant)
- Recommendations by parents to make the task more feasible for their child

For aim 2, results were explored in order to better understand the AS phenotype. The outcomes of the eye-tracking VOF task were the percentage of detected stimuli, fastest reaction time, and average reaction time. *Z*-scores were calculated using reference data from typically developing peers [36]. The eye-tracking social attention task was evaluated using the percentage of first looks to faces (attention capture) and total looks to faces (sustained attention). For fNIRS, oxy- and deoxygenated hemoglobin concentration changes of social versus non-social conditions were analyzed. The outcome of indirect calorimetry was the percentage difference between predicted and measured RMR, while the outcome of the BIA and BOD POD was percentage body fat. In addition, body mass index standard deviation scores (BMI SDS) were calculated. Appendix 3 gives a more elaborate description of the outcome variables and analyses used for each measurement instrument.

## Results

### Feasibility: success rate and data quality (aim 1)

**Fig. 2:**
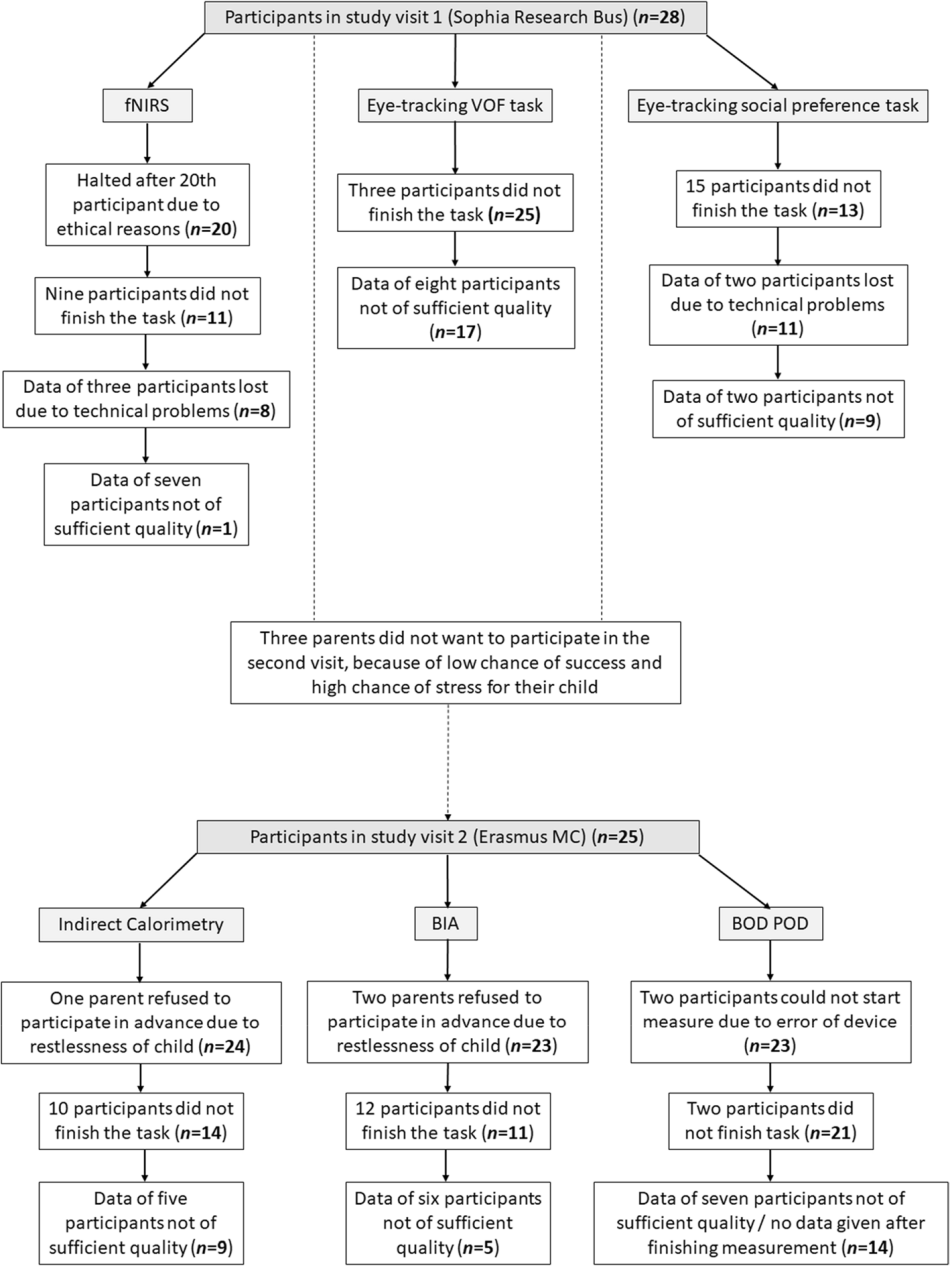
Flow chart of the feasibility of outcome measures

The feasibility of all outcome measures is depicted in Table 2 and Fig. 2. The eye-tracking VOF task and the BOD POD were found to be feasible outcome measures for children with AS. Both feasibility criteria were met: More than 70% of participants who started the measurement also finished the measurement (89 and 91%, respectively), and more than 60% of those who finished also contributed data of acceptable quality (68 and 67%, respectively). In contrast, the eye-tracking social preference task and the indirect calorimetry were not feasible, as not enough participants finished the measurement (46 and 58%, respectively). However, most participants who finished the measurements did have acceptable data quality (82 and 64%, respectively). BIA and fNIRS were not feasible for children with AS: Not enough participants finished the measurement (48 and 55%), and not enough participants contributed data of acceptable quality (45 and 13% - average Signal Quality Index = 2.42). Appendix 4 describes the feasibility per participant linked to genotype, age, sex, comorbid psychiatric diagnoses, medication use, vision, waking/standing abilities, and several measures of functioning. No clear association could be detected, except that most participants with UBE3A mutations successfully completed measures with acceptable data quality. Additional information on data quality is given in Appendix 5.

**Table 2:** Feasibility of outcome measures

	**Successfully finished/total *N* started**	**Data quality acceptable/total *N* finished**
Eye-tracking		
VOF task	**25/28 (89%)**	**17/25 (68%)**
Social preference task	13/28 (46%)	**9/11 (82%)**
fNIRS	11/20 (55%)	1/8 (13%)
Indirect calorimetry	14/24 (58%)	**9/14 (64%)**
Bio-impedance analysis	11/23 (48%)	5/11 (45%)
BOD POD	**21/23 (91%)**	**14/21 (67%)**

Explanation of differences in sample size is given in Fig. 2

*Abbreviations*: *VOF* Visual orienting functions, *fNIRS* Functional Near Infrared Spectroscopy, *BOD POD* whole body air-displacement plethysmography

The reasons for early termination of measurements are presented in Table 3. What stands out is that for the eye-tracking social preference task, failed calibration was the only reason participants could not finish the task. All participants that passed calibration were able to finish the task. Calibration failed because the child was not looking at the screen or the eye-tracker could not measure their gaze (for example, due to posture anomalies). In addition, indirect calorimetry finished early because participants pulled off the hood, or could not lay still for 20–30 min. During the BIA measurements, participants shook off the wires and clips. The fNIRS measurement was terminated early because participants pulled off the headcap and showed signs of distress. Four additional participants were able to finish the fNIRS measure, but did show significant signs of distress, which prevented them from paying attention to the screen. For all these participants, stress was a confounder that hindered us from measuring brain activity in response to social stimuli. Therefore, and in accordance with the code of conduct to the expression of objection by minors and people with intellectual disabilities, the fNIRS measurement was halted after the 20^th^ participant and outcomes were not analyzed.

**Table 3:** Reasons for early termination of measurements

	**Reasons for early termination of measurement***(% of all participants that started measurement)*
**Eye-tracking:**	
*- VOF task*	• Not paying attention to the screen (11%)
	• Not being able to sit still (7%)
	• Stress/anxiety (4%)
*- Social preference task*	• Failed calibration (54%)
**fNIRS**	• Pulling off the headcap (40%)
	• Showing signs of protest (crying, headbanging, hitting, kicking; 40%)
**Indirect calorimetry**	• Pushing off the hood or pulling of the hose (38%)
	• Not being able to lay still (33%)
	• Showing signs of protest (crying, kicking; 33%)
**BIA**	• Shaking off the wires or pulling loose the clips (43%)
	• Showing signs of protest (13%)
**BOD POD**	• Anxiety before going into the closed pod (9%)

### Feasibility: adaptations and ratings by parents (aim 1)

Table 4 describes adaptations made to the measurements. The most important adaptation was the use of the pre-set and post-calibration method for the eye-tracking VOF task. Twenty of 28 participants failed calibration at the first attempt (71%), after which the task was started using a pre-set calibration, and the data were calibrated after the measurement using a post-calibration method. This method entails scaling the center of the gaze positions to a corresponding target position (i.e., the center of the quadrant in which a stimulus is presented) [17]. For the eye-tracking social preference task, post-calibration was not possible due to software constraints and the nature of the task. Further adaptations were having participants sit in their own (wheel)chair, and providing distraction in the form of videos on a tablet. The BOD POD measurement was conducted without a swimming cap in some participants. The fNIRS measurement was started without fully optimizing light counts (removal of hair underneath the optodes).

**Table 4:** Adaptations made to the measurements

	**Adaptations made***(% of all participants that started measurement)*
**General adaptations** (eye-tracking and fNIRS test environment)	- Participants were encouraged to sit in their own (wheel)chair (to increase comfort and decrease motion) - Some participants were strapped into their chair belts by the parent (to decrease motion) - Some younger participants sat in their parents’ lap - Several parents held their child’s hands when performing the measurements (to prevent the child from pulling off the equipment)
**Eye-tracking:** - VOF - Social preference	- In case of failed calibration at first attempt, the task was started using a pre-set calibration, and the data were calibrated after the measurement using a post-calibration method (71%) - No adaptations made
**fNIRS**	- Measurement started without fully optimizing light counts (removal of hair underneath the optodes), because the participant showed signs of protest (35%) - Reversal of filming direction for co-registration (start at CZ and end in front of the face, as starting filming in front of the face led the participants to watch along the direction of the camera)
**Indirect calorimetry**	- Parents/caregivers held their child or their child’s hands (54%) - Distraction: videos and pictures were shown on the Ipad (42%) - A toy to hold in their hands (13%) - The measurement was done first on the parent to show the child how it works (4%) - The child was measured sleeping in his own stroller (4%)
**BIA**	- Distraction was provided to the child in the form of a video or by making funny faces - Pillows were put underneath arms and between legs to prevent touching (13%) - The clips were hidden underneath a bandage (4%) - Parents held their child with non-conducting material in between them (4%)
**BOD POD**	- The BOD POD has a scale on which participants have to stand still and balanced. Most children with AS could not do this. Therefore, their weight was assessed using a wheelchair scale, and manually inserted (61%) - Participant did not tolerate swimming cap, thus measurement was conducted without swimming cap (57%) - Calibrating Ipad or stuffed animal into the BOD POD (for distraction; 22%)

Acceptability and importance of the measurements/paradigms were rated by parents on a ten-point scale (see Table 5). The most acceptable measure was the BOD POD. Parents thought that this measure was experienced by their child as the most enjoyable (mean = 7.05, SD = 2.31) and least stressful (mean = 4.18, SD = 3.32). fNIRS was rated least enjoyable, with a mean grade of 3.33 (SD = 2.68), and most stressful, with a mean grade of 7.28 (SD = 2.20). All outcomes were considered important by parents (average grades 7.8 to 8.2). Recommendations given by parents to make the measurements more feasible are stated in Table 6. The most frequently mentioned recommendations were to make the stimuli more interesting and to skip calibration during the eye-tracking measurements. This could be achieved by using more attention-grabbing videos and sounds, personalizing the stimuli to the participant, and making the task interactive using a touchscreen.

**Table 5:** Acceptability and importance reported by parents on a ten-point scale. Reported values are mean (SD)

	**Eye-tracking**	**fNIRS**	**Indirect calorimetry**	**BIA**	**BOD POD**
**Q1.** How much do you think your child enjoyed the measurement?	5.48 (2.25)	3.33 (2.68)	5.67 (2.68)	4.89 (3.33)	7.05 (2.31)
**Q2.** How stressful do you think the measurement was for your child?	4.86 (2.69)	7.28 (2.20)	5.28 (3.49)	5.21 (3.30)	4.18 (3.32)
**Q3.** To what extend do you think this task measures an *important/relevant* aspect of your child’s functioning?	8.20 (1.70)	7.85 (2.45)	7.98 (1.33)	8.02 (1.54)	7.76 (1.54)

**Table 6:** Recommendations by parents to make the task more feasible for their child

	**Recommendations by parents to make the task more feasible for their child** *(suggestion given…times)*
Eye-tracking	- Make the stimuli more interesting (14x), for example by: • Using more attention-grabbing videos and sounds • Personalizing the stimuli to the participant • Making the task more interactive using a touch-screen - Skip or improve calibration (7x; e.g., by making the calibration target more interesting) - Improve the surroundings of the measurement (2x; as they found the Sophia Bus to be small and distracting)
fNIRS	- Make a practice cap that also includes the protruding tips of the optodes (2x; as this feeling was the most challenging for the participant) - Make the task stimuli more appealing to the participant (2x) - Do the measurement at home instead of in the Sophia Bus (1x) - Use a time timer (1x) - Allow the participant to stand instead of sit during the measurement (1x)
Indirect calorimetry	- Have a big TV screen on the ceiling with a distracting video (4x; instead of a video on a smaller Tablet as we did now) - Having a more comfortable lounge chair for the child to lay in (2x) - Having a smaller mask (2x) - Having a bigger mask (2x) - Give the participant more time to get used to the setting and measurement (1x) - Start the measurement right away with no delays (1x)
BIA	- Have a faster device (3x) - Most parents reported that this measurement was “just not possible” for their child
BOD POD	- Have distraction such as a video screen or buttons built into the BOD POD (4x) - Have a more comfortable seat with a seatbelt (3x) - Have relaxing music in the BOD POD (1x)

## Explorative results of outcome measures (aim 2)

### Eye-tracking visual orienting functions (VOF) task

Table 7 shows that participants with AS had exceptionally slow reaction times to high salient stimuli (cartoon and contrast) in comparison to typically developing peers, with average *Z*-scores ranging from 7.45 to 10.21. Of note, participants’ reaction times to low salient stimuli (color and global motion) were less different from their peers than reaction times to high salient stimuli, with *Z*-scores ranging from 0.26 to 2.48. In addition, what stands out in Table 7 is that high salient cartoons were detected less often (57%) than intermediate-salient forms (60%) and local motion patterns (71%).

**Table 7:** Eye-tracking visual orienting functions task: percentage detected stimuli and reaction times

**Stimulus**	**Detected**	**Fastest RT**		**Average RT**	
	**Stimuli (%)**	**Ms**	***Z*-score**	**Ms**	***Z*-score**
Cartoon	57	271	+ 7.45	320	+ 9.21
Contrast (100%)	71	459	+ 7.67	504	+ 10.21
Form	60	653	+ 6.98	711	+ 4.95
Local motion	71	627	+ 6.90	681	+ 8.13
Global motion	50	632	+ 2.04	713	+ 2.48
Color	21	898	+ 1.25	898	+ 0.26

### Eye-tracking social preference task

Only three participants had acceptable data for the assessment of attention capture (first looks) to faces. On average, participants dedicated their first fixations towards faces 41.67% of the time (SD = 38.19), but this was not significantly above the 20% chance level (*t* (2) = 0.98 and *p* = 0.215), likely due to the small sample size and large standard deviation. The analysis was repeated in a larger sample (*N* = 8) with data that was considered acceptable using only the second quality criterium (“moved their gaze to one of the five stimuli in the first three seconds of trial onset”; Appendix 2). Similar to the smaller group, participants had first fixations towards faces 41.46% of the time (SD = 16.94). This time, the difference to the 20% chance level was significant (*t* (7) = 3.58 and *p* = 0.004). Earlier studies using the same paradigm showed that typically developing infants had an average of 50.5% of first fixations towards faces [37].

Nine participants had acceptable data quality for the analysis of sustained attention (total fixation duration) to faces. The duration of fixations towards faces as a percentage of the total fixation duration (including non-social stimuli) was 22.88% (SD = 12.66), which did not differ significantly from the 20% chance level (*t* (8) = 0.68, *p* = 0.257). For comparison, typically developing infants dedicated around 41.5% of total fixations towards faces [37].

### Body mass index (BMI), indirect calorimetry, bio-impedance analysis (BIA), and BOD POD

Table 8 shows the results of the nineteen participants that successfully finished either the indirect calorimetry, BIA, or BOD POD measurement with data of acceptable quality. Their mean BMI SDS was 0.70 (SD = 1.62, range −1.52 to 3.36). Eleven participants were classified as having normal BMI (58%), five participants had overweight BMI (26%), and three participants had obese BMI (16%).

**Table 8:** BMI, BOD POD, bio-impedance analysis, and indirect calorimetry outcome per participant

**Genotype**	**Age**	**Sex**	**BMI**	**BMI**	**IC**	**IC Difference**^**a**^	**BIA**	**BIA**	**BOD POD**	**BOD POD**
			**(BMI SDS)**	**Category**	**Difference**^**a**^	**Category**	**PBF**	**PBF category**	**PBF**	**PBF category**
Deletion	2	Girl	14.63 (− 0.94)	Normal	− 13.11%	Fast	n.a	n.a	b.q	b.q
Deletion	8	Boy	13.93 (− 1.34)	Normal	n.a	n.a	n.a	n.a	19.70%	Normal
Deletion	9	Boy	17.75 (0.90)	Normal	− 8.00%	Normal	b.q	b.q	b.q	b.q
Deletion	9	Boy	15.38 (− 0.37)	Normal	n.a	n.a	n.a	n.a	18.00%	Normal
Deletion	12	Boy	15.12 (− 1.37)	Normal	b.q	b.q	b.q	b.q	25.40%	Overfat^+^
Deletion	12	Boy	21.78 (1.74)	Overweight	n.a	n.a	n.a	n.a	42.20%	Obese^+^
Deletion	13	Girl	20.67 (0.84)	Normal	b.q	b.q	n.a	n.a	44.70%	Obese^+^
Deletion	14	Boy	16.27 (− 1.52)	Normal	n.a	n.a	16.30%	Normal	n.a	n.a
Deletion	18	Girl	18.52 (− 1.19)	Normal	-23.00%	Slow	31.40%	Overfat^+^	37.40%	Obese^+^
ICD	8	Girl	17.09 (0.85)	Normal	n.a	n.a	n.a	n.a	24.10%	Normal
UPD	12	Girl	17.73 (− 0.08)	Normal	n.a	n.a	n.a	n.a	27.40%	Normal
UPD	13	Girl	31.4 (3.19)	Obese	10.10%	Normal	b.q	b.q	n.a	n.a
UPD	15	Boy	17.96 (− 1.14)	Normal	n.a	n.a	b.q	b.q	21.90%	Overfat^+^
UPD	17	Boy	25.6 (1.92)	Overweight	n.a	n.a	n.a	n.a	37.40%	Obese^+^
UBE3A mutation	6	Girl	18.24 (1.82)	Overweight	12.80%	Normal	30.90%	Obese^+^	n.a	n.a
UBE3A mutation	7	Boy	18.36 (1.88)	Overweight	2.60%	Normal	b.q	b.q	20.10%	Normal^−^
UBE3A mutation	9	Girl	20.22 (1.73)	Overweight	5.20%	Fast	b.q	b.q	23.30%	Normal^−^
UBE3A mutation	15	Boy	29.67 (2.98)	Obese	− 22.40%	Slow	35.70%	Obese	36.20%	Obese
UBE3A mutation	18	Girl	35.01 (3.36)	Obese	− 2.20%	Normal	51.10%	Obese	63.10%	Obese

*Abbreviations*: *BIA* Bio-impedance analysis, *BMI* Body mass index, *IC* Indirect calorimetry, *ICD* Imprinting center defect, *PBF* Percentage body fat, *UPD* Uniparental paternal disomy

*n.a.* measurement was not successfully finished

*b.q.* measurement was successfully finished, but with non-acceptable data quality

^a^Differences in percentage between predicted resting metabolism rate (Schofield; based on height and weight) and measured resting metabolism rate in kcal/day

^+^Classified one to two categories higher in PBF than in BMI

^-^Classified one category lower in PBF than in BMI

Nine participants had accurate data quality for indirect calorimetry. The mean difference (%) between the predicted and measured resting metabolism rate (RMR) was −1.32 (SD = 13.94), indicating that participants have 1.32% lower RMR than what would be expected based on their height and weight. However, the difference ranged from −23% to +13%. Two participants were classified as having hypometabolism, five had normal metabolism, and two had hypermetabolism.

For the BIA, five participants had data of acceptable quality. The mean percentage body fat (PBF) measured by the BIA was 33.08 (SD = 12.46). Fourteen participants had BOD POD data of acceptable quality. According to the BOD POD measurements, the mean PBF was 31.49 (SD = 12.71, range 18–63). For BOD POD and BIA, BPF was classified as “overfat/obese” in 57 to 80% of participants, respectively, in comparison to typically developing peers [38]. In addition, the amount of body fat was higher than what would be expected based on the BMI SDS in approximately 40% of participants.

## Discussion

The current study investigated the feasibility and explored the results of five innovative candidate outcome measures on neurocognitive functioning and physical growth for children with AS, namely, eye-tracking (VOF task and social preference task), functional Near Infrared Spectroscopy (fNIRS), indirect calorimetry, bio-impedance analysis (BIA), and BOD POD. Results show that the eye-tracking VOF task and the BOD POD were feasible in children with AS, as approximately 90 percent finished the measurements and approximately 65 percent of them contributed data of acceptable quality. In contrast, not enough participants were able to finish the eye-tracking social preference task (46%) and the indirect calorimetry measurement (58%), but those who finished generally did have data of acceptable quality (82% and 64%). fNIRS and BIA were not feasible for children with AS, as not enough participants finished the measurements (55% and 48%), and the data were not of acceptable quality (13% and 45%). The most common reasons for early termination of measurements were failed calibration during eye-tracking and participants showing signs of protest during fNIRS, BIA, and indirect calorimetry. The most important adaptation made to our measurement was using a pre-set and post-calibration method for eye-tracking. Parents rated the BOD POD as the most acceptable measure for their child, while fNIRS was reported to be the least acceptable. All outcomes were rated as important by parents. Exploratory outcomes of the eye-tracking VOF task indicated that children with AS have longer reaction times in detecting high salient visual stimuli than typically developing peers. Eye-tracking further showed that social stimuli (faces) captured participants’ attention considerably more often than non-social stimuli, but sustained attention was not different for social versus non-social stimuli. Concerning body composition, a high proportion of fat was measured in 57–80 percent of participants. For approximately 40 percent of participants, the amount of body fat was higher than what would be expected based on the BMI SDS.

Our finding that the eye-tracking VOF task was feasible for children with AS, but the eye-tracking social preference task was not, highlights the importance of a good calibration procedure. The eye-tracking VOF task had the possibility for post-calibration, while the social preference task did not. Previous studies on eye-tracking in AS also reported calibration difficulties as the main reason for the early termination of the measurement [13, 14]. Differences in calibration procedure may explain the varying success rates of 47 to 83 percent reported in earlier studies [13–15]. We propose that the possibility of adapting the calibration procedure to the AS group, in our case by means of a post-calibration method, is crucial for the success of the measurement. Parents suggested that further improvement of eye-tracking tasks for children with AS could be achieved by incorporating more attention-grabbing videos and sounds, personalizing stimuli to the participant, and making the task interactive using a touch-screen.

Importantly, our finding that the BOD POD is a feasible outcome measure for children with AS emphasizes the fact that feasibility cannot always be predicted in advance. Although the BOD POD is proven a more precise and reliable technique than BIA in obese and non-obese children [39], the BOD POD requires participants to sit still in a small closed pod for 3 min in their underwear with a swimming cap on, making it prone to participant anxiety and discomfort. We expected the BOD POD to be less feasible than BIA, but were surprised by the success rate and acceptability of the BOD POD.

Finally, fNIRS, BIA, and indirect calorimetry were not feasible in their current form for most children in our sample. One similarity between these measurements is that they all involve the attachment of a measurement instrument (headcap, clips, or mask) to the participant’s body. Earlier studies indicated that individuals with AS show particular signs of hyper-responsiveness to tactile stimuli, with 64% showing distress while being cared for [40]. These findings suggest that instruments involving any pressure on the body may be less suitable for children with AS. However, we appreciate the possibility that more extensive practice with the fNIRS cap or testing a group with other patient characteristics (e.g., older individuals with less behavioral issues) might yield better results in terms of feasibility. In addition, for those participants that did endure the indirect calorimetry measure, data quality was sufficient, and results were usable.

Exploring the outcome of the eye-tracking VOF task, we saw that children with AS have longer reaction times in detecting high salient visual stimuli. In line with this finding is the knowledge that children with intellectual disabilities have slower information processing speed [41]. It also corroborates recent findings from another AS eye-tracking study that found delayed reaction times in detecting a named object visually, which they attributed to delayed language processing and attention problems [15]. A surprising finding of the current study was that participants’ reaction times to low salient stimuli were less different from their peers than their reaction time to high salient stimuli. This could be because high salient stimuli elicit the fastest possible reaction time, while reaction times to low salient stimuli show more variation and stabilize at a higher age than reaction times to high salient stimuli [36].

The eye-tracking social preference task indicated that faces capture attention in children with AS more than non-social objects, similarly as in typically developing peers. Previous studies have reported high prevalence of autism symptoms in AS [42], whereas other studies reported high sociability [43, 44]. The current finding might be interpreted as evidence for high sociability. On the other hand, lower attention capture of faces in children with ASD has been found in some studies using the same paradigm [45], but not all [37], indicating that this may not be a robust early marker of autism. In contrast to our first finding of normal attention capture for social stimuli in AS, our second finding indicated that sustained attention (total fixation duration) was not higher for social stimuli than for non-social stimuli. This could indicate less preference for social stimuli in AS, consistent with other studies focusing on sustained attention to social stimuli [13, 14]. Finally, children with high autistic features may have been less likely to participate in the current study than children with low autistic features. Replication in a larger sample is required.

With respect to body composition as measured by the BOD POD, we found high percentages of body fat in comparison to typically developing peers in 57% of participants and percentages of body fat that were higher than expected on the basis of BMI SDS in 43% of participants. From earlier studies, we know that overweight and obesity is more prevalent in children with AS compared to their neurotypical peers [29, 46] and that reinstatement of UBE3A reverses overweight in an AS mouse model [30, 31]. An explanation for the fat percentage being higher than expected on the basis of BMI may be that some children with AS have lower muscle mass [47]. Of note, seven out of 28 of the children in our study sample have AS due to the UPD genotype (25%). This is higher than in the general AS population, were 5 to 10% present with the UPD genotype [2]. Some studies indicate that children with the UPD genotype have a higher BMI than children with AS due to other genotypes [48], although other studies do not confirm this difference [49]. We recommend future studies to investigate body composition in a larger sample.

Strengths of the current study are the use of innovative outcome measures that are largely new in AS research. In addition, using our mobile research lab in a camper van made it possible to visit participants close to their home. Participants not needing to travel minimized the burden of participation, as well as fatigue and sensory overload at the moment of testing, while still having the benefit of a standardized and quiet testing environment. Further strong points are the involvement of parents who gave recommendations and rated acceptability, the preparation of participants with pictograms and practice caps, and the flexibility induced by allowing for adaptations to measurements along the way. A major limitation of this study is the potential bias in our research sample. Eleven parents stated that they did not want to participate because they thought the chances of success were small or the measures may induce stress on their child. Possibly, this causes a bias in results in the direction of overly positive feasibility findings. Nevertheless, participants did not differ from non-participants on age, sex, or genotype. In addition, having an eye-tracker on an arm that is rotatable in three dimensions was not possible in our mobile set up, which made it impossible to account for all posture variations in children with AS. Also, in the ASD literature, the use of a static social preference paradigm for eye-tracking less consistently elicits differences in social preference between ASD groups and control groups than the use of a complex social video with an interesting non-social background [50]. Although the current simple and short paradigm served like a good “proof of principle” for the feasibility of eye-tracking in AS, future studies should consider using a dynamic paradigm. Lastly, the current study did not aim to measure construct validity, as there are no golden standards or existing measures of the same construct in the AS population, and considering our small sample size. We recommend future studies to broadly investigate the possible correlation between the measures found feasible in this study and measures of related constructs.

The findings of this study have important practical implications, stated in Table 9. In addition, we propose that eye-tracking is a promising measurement instrument for children with AS when using alternative or adaptable calibration methods. Task stimuli should include attention-grabbing movies and sounds and ideally be adaptable to age and personal preferences of the participant. The eye-tracker arm should be rotatable in three dimensions to account for all posture variations in AS. Eye-tracking can potentially be used to measure a variety of outcomes, such as basic visual functions, social preference, or understanding of language. Exploratory outcomes showed longer reaction times to visual stimuli. This indicates that when offering visual information to a child with AS, researchers, parents, or teachers should give them more time to process the information and produce a response. In addition, the BOD POD is a feasible way to measure body composition in children with AS and is suitable for use in clinical practice and research. We measured high levels of body fat in children with AS, also when the BMI was normal. These findings warrant further (larger) studies on growth and metabolism in this population and suggest that clinical nutritional assessment should receive attention in clinical practice. Body composition and metabolism could serve as additional outcome measures for future therapy trials, as restoring UBE3A function leads to reversal of increased body weight in AS mice [30, 31].

**Table 9:** Practical implications

**Ideal outcome measures for children with Angelman syndrome:**
**Should not:**
⨂ Rely on language or motor functioning
⨂ Require a certain level of intellectual functioning
⨂ Touch the body of the child
**Should:**
✓ Be short
✓ Take place in a distraction-free environment
✓ Allow the child to move (slightly)
✓ Involve the parent/caregiver
✓ Use interesting task stimuli or distractions
✓ Take into account longer reaction times

## Conclusions

This study shows that eye-tracking and BOD POD are feasible outcome measures for children with AS. These measures offer potential for clinical practice, research, and medication trials. fNIRS, bio-impedance analysis, and indirect calorimetry were not feasible for children with AS. Exploring the results of the eye-tracking measurements showed longer reaction times to salient visual stimuli. Concerning body composition, exploratory results demonstrated a high percentage of body fat in the majority of participants, indicating that the assessment of weight and body composition should receive attention.

## Data Availability

The datasets used and/or analyzed during the current study are not publicly available due to privacy/ethical reasons, but are available from the corresponding author on reasonable request.

## Appendix 1

**Table 10:** Additional information on eye-tracking and fNIRS measures

**Measure**	**Description**
Eye-tracking	Participants sat approximately 65-cm distance to the screen. Sampling frequency was 300 Hz for part of the sample (37–42%) and 60 Hz for another part of the sample (58–63%). A webcam recorded the child’s behavior during the test assessment
- VOF task	Each stimulus is presented in one of four quadrants on the screen, while the other quadrants remain empty. According to the principle of preferential looking, if a child sees the stimulus, it will look at it
- Social preference task	Participants were presented a ring of five stimuli, containing an image of a face with direct gaze, a scrambled inverted face (noise face), and three non-social stimuli (car, phone, and bird). The current experiment used six slides for a duration of 10 s each. A fixation stimulus (cartoon) was presented in the center of the screen for three seconds in between slides
fNIRS	The Artinis Brite 23 was originally developed for use on the frontal regions, but was used in this study on the Superior Temporal Sulcus (STS) region with custom made headcaps utilizing 18 channels. We obtained good signal using this setup in healthy subjects. The system used two wavelengths of emitting light at 760 and 850 nm. Source-detector separation was 25 mm for cap sizes 46 to 50 cm and 30 mm for cap sizes 52 to 58 cm. Participants were given the option to watch an entertaining video, while the headcap was placed on the head. The task entailed a visual-social condition, non-vocal condition, and vocal condition, displayed in a repeating loop six times (total task duration approximately 6 min). In between the different conditions was a baseline period, which consisted of visual non-social stimuli (pictures of vehicles). The visual social dynamic stimuli involved videos of a person who either moved her eyes left or right or performed hand games (“peek-a-boo” and “incy wincy spider”). The non-vocal condition consisted of environmental sounds (running water, squeaky toys), while the vocal condition contained non-language vocalizations, such as laughing and yawning

## Appendix 2

**Table 11:** Methods for data quality assessment

**Measure**	**Data quality assessment**
Eye-tracking	
- VOF task	Data quality was manually inspected by looking at the amount of gaze data points present (*X*- and *Y*-coordinates of at least one eye) and the amount of gaze data points directed to a stimulus (in contrast to undirected gaze data points). Data was considered of acceptable quality when post-calibration was possible. For informative purpose, data loss and accuracy were reported. Criteria to determine if a stimulus has been seen and to determine reaction time can be found in Kooiker et al. [16]
- Social preference task	A slide was considered valid to assess attention capture (first looks) to faces when participants were fixated at the center of the screen in the last 500 ms before trial onset and fixated on an Area Of Interest (AOI) in the first three seconds after trial onset. A trial was considered valid to assess sustained attention (total fixation duration) to faces when the participant spent longer than one second fixating AOIs on the slide (based on criteria from previous literature using the same task) [19, 37, 51, 52]. Acceptable data quality was defined as having at least three valid trials for either the attention capture or sustained attention analyses. Data loss (%), precision (noise level), and accuracy were reported for informative purpose
fNIRS	Data quality was visually inspected and graded per channel per participant. In addition, the signal quality index (SQI)—an algorithm for quantitative assessment of fNIRS signal quality—was calculated per channel. Both the manual grades and SQI ranged from zero (very bad quality) to five (very good quality). Participants were considered to have acceptable data quality when the average of the visually inspected grade and SQI was 3, 5 or higher [53]
Indirect calorimetry	Data quality was considered acceptable if a steady-state ventilation of at least 5 min was reached, and when minute-to-minute oxygen consumption and carbon dioxide elimination varied by ≤ 10%. The first 5 min of a measurement were not taken into account, because the RMR is known to decline and then stabilize due to acclimatization of the participant. These criteria were based on previous literature [54–56]. In addition, data quality was considered unacceptable if the result was clinically impossible (e.g., a difference between measured and predicted RMR of 60%)
BIA	Data were considered of unacceptable quality when the BIA showed the notification “Perform the measurement again. Incorrect posture may cause inaccurate results,” which was displayed in case two or more impedance values were reversed, in case of high impedance values in the trunk, and in case of big impedance drops. This can be caused by movement of the child, when the segments are not separated from each other (e.g., arms touched the torso), or when the parent touched the child. In addition, the quality was considered unacceptable when the outcome was clinically impossible (e.g., extremely low fat percentage in combination with high BMI)
BOD POD	Data were considered of unacceptable quality when the outcome was clinically impossible, for example, extremely low fat levels in combination with a high BMI. Further quality measures were not available for the BOD POD

## Appendix 3

**Table 12:** Outcome variables and analysis methods

**Measure**	**Outcome variables and analysis**
Eye-tracking	
- VOF task	Outcomes were percentage of detected stimuli, fastest reaction time, and average reaction time. The AOI consists of a circle with a 6 to 8 degree radius (depending on the size of the stimulus). A detailed description of criteria to determine if a stimulus was detected and to determine reaction time can be found in Kooiker et al. [16]. Participants were compared to typically developing children of their closest age (reference groups available until 12 years old) [16], and *Z*-scores were calculated
- Social preference task	Outcomes were percentage of first looks (attention capture) and total looks (sustained attention) towards faces. To evaluate whether participants directed their first and total looks more often towards faces than would be expected by chance (20%), a one sample *t*-test was conducted. To identify fixations, identification by 2-means clustering was used, an algorithm built specifically for data across a wide range of noise levels [57]. Fixations were then assigned to an area of interest (AOI) using the Limited-Radius Voronoi Tessellation method [58] with a radius of four degrees
fNIRS	Oxy- and deoxygenated hemoglobin concentration changes were calculated. Signals were filtered to eliminate task-irrelevant systemic physiological oscillations. Data that were considered unusable (e.g., due to extreme motion) were excluded from analyses. The visual social condition was contrasted to the baseline, and the vocal condition was contrasted to the non-vocal condition. In this manner, the hemodynamic response was specific to the social stimuli, rather than visual or auditory stimulation per se
Indirect calorimetry	Resting metabolism rate (RMR) was predicted on the basis of height and weight [59]. The percentage difference between predicted and measured RMR was calculated. This difference was classified into slow, normal or fast metabolism
BIA and BOD POD	Percentage of body fat was used as outcome for both BIA and BOD POD and was classified into categories (underfat/normal/overfat/obese) using reference curves for children [38]. Lohman density model was used for BOD POD measurements
Body mass index (BMI)	BMI and BMI standard deviation score (SDS) were calculated using the pediatric formula of the Netherlands Organization for Applied Natural Science Research. BMI was categorized into underweight (≤ − 2 SDS), normal weight (> − 2 and < 1 SDS), overweight (≥ 1 SDS), or obese (≥ 2 SDS) using cut-off points determined by the World Health Organisation

## Appendix 4

**Table 13:** Description of feasibility linked to phenotypical information: part A

**Genotype**	**Age**	**Sex**	**Eye-tracking tasks**		**fNIRS**	**Indirect calorimetry**	**BIA**	**BOD POD**	**Clinical Global Impression of Severity**^**f**^				
			**VOF**	**Social pref**					**Total**	**Behavior**	**Motor**	**Communication**	**Sleep**
											**Fine/gross**	**Exp./Recept**	
Deletion	2	Boy	No	Yes, Q+	Yes, Q-	Yes, Q-	No	Yes, Q-	5	4	4/3	5/4	5
Deletion	2	Girl	Yes, Q-	Yes, Q-	Yes, Q-	Yes, Q+	No	Yes, Q-	4	4	4/5	6/5	4
Deletion	6	Boy	Yes, Q+	No	No	Refused^c^	Refused^c^	Yes, Q-	5	5	5/4	5/5	6
Deletion	6	Girl	Yes, Q+	Yes, Q+	No	Yes, Q-	No	Yes, Q-	5	3	6/6	6/5	4
Deletion	8	Boy	Yes, Q+	No	No attempt^b^	No	No	Yes, Q+	5	5	5/4	6/4	2
Deletion	8	Girl	Yes, Q+	No	No attempt^b^	No	No	Yes, Q-	6	3	6/5	7/6	2
Deletion	9	Boy	No	No	No attempt^b^	No	No	Yes, Q+	4	3	5/3	5/3	2
Deletion	9	Boy	Yes, Q-	No	No	Yes, Q+	Yes, Q-	Yes, Q-	5	3	5/5	6/4	3
Deletion	12	Boy	Yes, Q+	Yes, Q+	No	No	No	Yes, Q+	6	5	5/6	6/4	1
Deletion	12	Boy	Yes, Q-	Yes, Q+	No attempt^b^	Yes, Q-	Yes, Q-	Yes, Q+	6	3	5/6	7/6	2
Deletion	12	Boy	Yes, Q+	No	No	Refused^d^	Refused^d^	Refused^d^	4	6	4/3	5/2	3
Deletion	12	Girl	No	Yes, Q+	No	Refused^d^	Refused^d^	Refused^d^	6	3	5/6	6/5	3
Deletion	13	Girl	Yes, Q+	No	No	Yes, Q-	No	Yes, Q+	5	5	5/6	4/2	3
Deletion	14	Boy	Yes, Q-	Yes, Q-	Yes, Q+	Yes, Q-	Yes, Q+	No attempt^e^	5	3	5/6	5/3	1
Deletion	18	Girl	Yes, Q+	No	Yes, Q-	Yes, Q+	Yes, Q+	Yes, Q+	5	3	5/5	3/2	5
UPD	7	Girl	Yes, Q-	No	Yes, Q-	No	No	No	3	3	4/3	3/2	5
UPD	8	Girl	Yes, Q-	No	No	Refused^d^	Refused^d^	Refused^d^	4	5	4/4	5/3	3
UPD	12	Girl	Yes, Q-	Yes, Q?^a^	Yes, Q?^a^	No	No	Yes, Q+	4	5	4/3	4/3	4
UPD	13	Girl	Yes, Q+	No	Yes, Q?^a^	Yes, Q+	Yes, Q-	No attempt^e^	5	4	5/5	5/4	1
UPD	14	Boy	Yes, Q+	Yes, Q+	No	No	Refused^c^	No	4	4	4/3	4/2	5
UPD	15	Boy	Yes, Q+	No	No attempt^b^	No	Yes, Q-	Yes, Q+	4	4	5/4	4/3	5
UPD	17	Boy	Yes, Q +	No	No attempt^b^	No	No	Yes, Q+	4	5	5/6	4/3	2
ICD	7	Girl	Yes, Q+	Yes, Q?^a^	Yes, Q?^a^	No	No	Yes, Q+	5	6	4/3	4/3	6
Mutation	5	Girl	Yes, Q-	No	No attempt^b^	Yes, Q+	Yes, Q+	Yes, Q-	4	3	4/2	4/3	5
Mutation	7	Boy	Yes, Q+	Yes, Q+	No attempt^b^	Yes, Q+	Yes, Q-	Yes, Q+	4	4	4/3	5/3	3
Mutation	9	Girl	Yes, Q+	No	Yes, Q-	Yes, Q+	Yes, Q-	Yes, Q+	4	4	5/4	4/3	1
Mutation	15	Boy	Yes, Q+	Yes, Q+	Yes, Q-	Yes, Q+	Yes, Q+	Yes, Q+	5	4	4/2	5/3	6
Mutation	18	Girl	Yes, Q+	Yes, Q+	Yes, Q-	Yes, Q+	Yes, Q+	Yes, Q+	5	3	5/5	5/3	5

*Abbreviations*: *BIA* Bio-impedance analysis, *BOD POD* whole body air-displacement plethysmography, *Exp.* Expressive communication, *fNIRS* Functional Near Infrared Spectroscopy, *ICD* Imprinting center defect, *Recept.* Receptive communication, *Social pref.* Social preference task, *UPD* Uniparental paternal disomy, *VOF* Cerebral visual impairment task, *Q+* Acceptable data quality, *Q-* Non-acceptable data quality

^a^Quality not assessable due to technical problems

^b^fNIRS measurement halted after 20th participant due to ethical reasons

^c^Child or legal representative refused to participate in this specific measurement beforehand

^d^Child of legal representative refused to participate in second study visit

^e^Measurement could not be started due to technical problems

^f^The CGI was adapted to the AS population by the authors. A score of 1 represents normal development, 2 is slightly impaired, 3 is mildly impaired, 4 represents moderately impaired, 5 is markedly impaired, 6 is severely impaired, and 7 represents very severe impairments (among the most extremely impaired)

**Table 14:** Description of feasibility linked to phenotypical information: part B

**Genotype**	**Age**	**Sex**	**Eye-tracking tasks**		**fNIRS**	**Indirect calorimetry**	**BIA**	**BOD POD**	**PEDI-CAT scaled score (*****T*****-score)**^**f**^		
			**VOF**	**Social pref**					**Daily activities**	**Mobility**	**Social/cognitive**
Deletion	2	Boy	No	Yes, Q+	Yes, Q-	Yes, Q-	No	Yes, Q-	42 (35)	55 (41)	47 (29)
Deletion	2	Girl	Yes, Q-	Yes, Q-	Yes, Q-	Yes, Q+	No	Yes, Q-	43 (29)	56 (34)	51 (29)
Deletion	6	Boy	Yes, Q+	No	No	Refused^c^	Refused^c^	Yes, Q-	47 (15)	60 (23)	52 (< 10)
Deletion	6	Girl	Yes, Q+	Yes, Q+	No	Yes, Q-	No	Yes, Q-	42 (< 10)	52 (< 10)	49 (< 10)
Deletion	8	Boy	Yes, Q+	No	No attempt^b^	No	No	Yes, Q+	-	-	-
Deletion	8	Girl	Yes, Q+	No	No attempt^b^	No	No	Yes, Q-	-	-	-
Deletion	9	Boy	No	No	No attempt^b^	No	No	Yes, Q+	-	-	-
Deletion	9	Boy	Yes, Q-	No	No	Yes, Q+	Yes, Q-	Yes, Q-	-	-	-
Deletion	12	Boy	Yes, Q+	Yes, Q+	No	No	No	Yes, Q+	-	-	-
Deletion	12	Boy	Yes, Q-	Yes, Q+	No attempt^b^	Yes, Q-	Yes, Q-	Yes, Q+	-	-	-
Deletion	12	Boy	Yes, Q+	No	No	Refused^d^	Refused^d^	Refused^d^	50 (< 10)	62 (< 10)	54 (< 10)
Deletion	12	Girl	No	Yes, Q+	No	Refused^d^	Refused^d^	Refused^d^	-	-	-
Deletion	13	Girl	Yes, Q+	No	No	Yes, Q-	No	Yes, Q+	46 (< 10)	52 (< 10)	49 (< 10)
Deletion	14	Boy	Yes, Q-	Yes, Q-	Yes, Q+	Yes, Q-	Yes, Q+	No attempt^e^	39 (< 10)	44 (< 10)	50 (< 10)
Deletion	18	Girl	Yes, Q+	No	Yes, Q-	Yes, Q+	Yes, Q+	Yes, Q+	49 (< 10)	56 (< 10)	60 (< 10)
UPD	7	Girl	Yes, Q-	No	Yes, Q-	No	No	No	50 (22)	60 (19)	57 (12)
UPD	8	Girl	Yes, Q-	No	No	Refused^d^	Refused^d^	Refused^d^	50 (14)	59 (< 10)	54 (< 10)
UPD	12	Girl	Yes, Q-	Yes, Q?^a^	Yes, Q?^a^	No	No	Yes, Q+	51 (< 10)	63 (< 10)	56 (< 10)
UPD	13	Girl	Yes, Q+	No	Yes, Q?^a^	Yes, Q+	Yes, Q-	No attempt^e^	44 (< 10)	52 (< 10)	51 (< 10)
UPD	14	Boy	Yes, Q+	Yes, Q+	No	No	Refused^c^	No	51 (< 10)	65 (< 10)	58 (< 10)
UPD	15	Boy	Yes, Q+	No	No attempt^b^	No	Yes, Q-	Yes, Q+	48 (< 10)	59 (< 10)	51 (< 10)
UPD	17	Boy	Yes, Q+	No	No attempt^b^	No	No	Yes, Q+	-	-	-
ICD	7	Girl	Yes, Q+	Yes, Q?^a^	Yes, Q?^a^	No	No	Yes, Q+	51 (24)	62 (26)	56 (< 10)
Mutation	5	Girl	Yes, Q-	No	No attempt^b^	Yes, Q+	Yes, Q+	Yes, Q-	49 (24)	64 (38)	53 (< 10)
Mutation	7	Boy	Yes, Q+	Yes, Q+	No attempt^b^	Yes, Q+	Yes, Q-	Yes, Q+	45 (< 10)	61 (20)	51 (< 10)
Mutation	9	Girl	Yes, Q+	No	Yes, Q-	Yes, Q+	Yes, Q-	Yes, Q+	51 (13)	62 (< 10)	63 (22)
Mutation	15	Boy	Yes, Q+	Yes, Q+	Yes, Q-	Yes, Q+	Yes, Q+	Yes, Q+	56 (< 10)	63 (< 10)	62 (< 10)
Mutation	18	Girl	Yes, Q+	Yes, Q+	Yes, Q-	Yes, Q+	Yes, Q+	Yes, Q+	51 (< 10)	61 (< 10)	60 (< 10)

*Abbreviations*: *BIA* Bio-impedance analysis, *BOD POD* Whole body air-displacement plethysmography, *fNIRS* Functional Near Infrared Spectroscopy, *ICD* Imprinting center defect, *PEDI-CAT* Pediatric Evaluation of Disability Inventory Computer Adaptive Test, *Social pref.* Social preference task, *UPD* Uniparental paternal disomy, *VOF* Cerebral visual impairment task, *Q+* Acceptable data quality, *Q-* Non-acceptable data quality

^a^Quality not assessable due to technical problems

^b^fNIRS measurement halted after 20^th^ participant due to ethical reasons

^c^Child or legal representative refused to participate in this specific measurement beforehand

^d^Child of legal representative refused to participate in second study visit

^e^Measurement could not be started due to technical problems

^f^Scaled scores on the PEDI-CAT lay on a continuum of 20 (low function) to 80 (high function) and are not age-related. *T*-scores were not reported, as more than half of the population had a *T*-score lower than 10, under which no differentiation was possible

## Appendix 5

This appendix gives additional information on the quality of the eye-tracking tasks.

**Table 15:** Additional data quality measures

**Measure**	**Data quality**
Eye-tracking	
- VOF task	For the participants that passed (post)calibration, data loss ranged from 43 to 58% depending on stimulus type. Accuracy was 3.16 degrees visual angle on average (SD = 1.17) for the cartoon stimulus, although it has to be noted that the cartoon stimulus has a large AOI and moved to attract attention, which can explain the accuracy being larger
- Social preference task	The average amount of data loss was 43%, while the mean precision (RSM noise level) was 1.26 (SD = 0.69). Accuracy was 0.90 degrees visual angle on average (SD = 0.38) Although the amount of data loss is high, previous studies reported that high amounts of data loss do not necessarily mean that data is not useful [60]

## Abbreviations

ADHD: Attention-deficit/hyperactivity disorder
AOI: Area of interest
AS: Angelman syndrome
ASD: Autism spectrum disorder
BIA: Bio-impedance analysis
BMI: Body mass index
fNIRS: Functional Near-Infrared Spectroscopy
IC: Indirect calorimetry
ICD: Imprinting center defect
PBF: Percentage body fat
RMR: Resting metabolism rate
ROSA: Rotterdam outcome study for children with Angelman syndrome
SDS: Standard deviation score
SQI: Signal quality index
(UBE3A) mutation: Pathogenic variant of the UBE3A gene
UPD: Uniparental paternal disomy
VOF: Visual orienting functions
